# Smallpox Still Haunts Scientists: Results of a Questionnaire-Based Inquiry on the Views of Health Care and Life Science Experts and Students on Preserving the Remaining Variola Virus Stocks

**DOI:** 10.1155/2013/672813

**Published:** 2013-07-22

**Authors:** Thangavelu Srinivasan, Vidyasagar Devaprasad Dedeepiya, Sudhakar John, Rajappa Senthilkumar, Helen C. Reena, Paramasivam Rajendran, Madasamy Balamurugan, Gene Kurosawa, Masaru Iwasaki, Senthilkumar Preethy, Samuel J. K. Abraham

**Affiliations:** ^1^The Fujio-Eiji Academic Terrain (FEAT), Nichi-In Centre for Regenerative Medicine (NCRM), PB 1262, Chennai 600034, India; ^2^The Mary-Yoshio Translational Hexagon (Myth), Nichi-In Centre for Regenerative Medicine (NCRM), PB 1262, Chennai 600034, India; ^3^Sri Ramachandra Medical College and Research Institute, Sri Ramachandra University, Sri Ramachandra Nagar, Porur, Chennai 600116, India; ^4^Ruma Biotherapy Research Centre, 2nd No. 21.A1 Ground Floor, Block C, Shanthi Apartments, 1st Cross Street, TTK Road, Alwarpet, Chennai 600018, India; ^5^Institute for Comprehensive Medical Science, Fujita Health University, 1-98 Dengakugakubo, Kutsukake-cho, Toyoake, Aichi 470-1192, Japan; ^6^School of Medicine, Yamanashi University, 1110 Shimokato, Yamanashi, Chuo 409-3898, Japan; ^7^Hope Foundation (Trust), B6, 13 Zakariah Colony III Street, Choolaimedu, Chennai 600094, India

## Abstract

The World Health Organization (WHO) declared eradication of the dreadful disease “smallpox” in 1980. Though the disease has died down, the causative virus “variola” has not, as it has been well preserved in two high security laboratories—one in USA and another in Russia. The debate on whether the remaining stocks of the smallpox virus should be destroyed or not is ongoing, and the World Health Assembly (WHA) in 2011 has decided to postpone the review on this debate to the 67th WHA in 2014. A short questionnaire-based inquiry was organized during a one-day stem cell meeting to explore the views of various health care and life science specialists especially students on this aspect. Among the 200 participants of the meeting, only 66 had answered the questionnaire. 60.6% of participants who responded to the questionnaire were for preserving the virus for future reference, while 36.4% of the participants were for destroying the virus considering the magnitude with which it killed millions. However, 3% of the respondents were not able to decide on any verdict. Therefore, this inquiry expresses the view that “what we cannot create, we do not have the right to destroy.”

## 1. Introduction

Smallpox was once a rampant and devastating disease in several parts of the world [1]. The World Health Organization (WHO) initiated the eradication programme in 1958 and intensified it since January 1967. The WHO declared eradication of smallpox by a resolution adopted in the World Health Assembly (WHA), the resolution WHA 33.3 on 8th May 1980 [2]. The last naturally occurring case was reported in Somalia on 26th October 1977 [3]. It is assumed that about 300 million people died of smallpox in the twentieth century alone. Approximately 30% of those infected with smallpox died around the globe and those who survived lived with ugly scars.

In the posteradication era, the resolution WHA 33.4 directed that all variola viruses globally were to be destroyed, except at two international centres, one in USA and another in USSR. The WHA Resolution 49.10 recommended that all existing smallpox virus stocks were to be destroyed on June 30, 1999. However, the same was not carried out, and in the resolution WHA 60.1 the WHA requested the Director General to undertake a major review in 2010 on the research previously undertaken, those that were underway, and the plans and requirements for further essential research so that the Sixty-fourth World Health Assembly in 2011 might reach a global consensus on the timing of the destruction of existing variola virus stocks [4, 5]. During the meeting of WHA in May, 2011, it was decided to postpone the review on this aspect to 67th WHA in 2014 [6]. So we still have reasons to fear smallpox as there are at present 450 isolates of variola preserved in the WHO collaborating centre in the United States [7] and 691 samples preserved in the WHO collaborating centre in Russia [8] signalling the chance of inappropriate release of live virus from these high security laboratories at any time.

Therefore, the debate on the logic of retaining or destroying stocks of smallpox is haunting the minds of scientists as well as the World Health Assembly (WHA) [9]. With this background, the lead authors of this paper decided to conduct an inquiry on this aspect among the clinicians and researchers who attended a stem cell meeting in a developing Asian nation and bring out their verdict on destruction or continued preservation of variola virus stocks.

## 2. Materials and Methods

### 2.1. Description of the Event and Participants of the Survey

The participants of the inquiry comprised a rare blend of research scholars, students, and clinicians from biotechnology, life sciences, medicine, veterinary, and dentistry who attended a one-day stem cell meeting conducted on 15th October in 2011. The meeting had a quiz, a symposium, and plenary lectures as various components [10]. The total number of participants were 200, out of which 23.93% were students in faculty of medicine, 61.59% were from the field of biotechnology and allied basic sciences, 11.59% were from dentistry, and 2.89% were from veterinary sciences. 74.31% of the meeting participants were students from the various fields mentioned above. Majority of the attendees (96–98%) were from India while the rest (2–4%) were from countries like Japan, Canada, and Malaysia.

The inquiry was conducted by a questionnaire (Table 1) distributed to the participants. A brief history of smallpox, the current topic of debates whether to preserve or to destroy smallpox, its importance in terms of biological research, and the potential threats were explained by an expert (one of the coauthors of this paper—Dr. Sudhakar John) who has a teaching experience of more than a decade in community ophthalmology and also had a fellowship training in the London School of Tropical Medicine. The participants were also allotted adequate time to interact and clarify their doubts with the expert. At the end, they were given 20 minutes to fill the form (Table 1), concurrently allowing them to interact with the peer group and also other experts who had gathered for the stem cell meeting. The questionnaire had totally 10 questions with ample space provided to express their views on the topic of whether the smallpox virus stocks should be destroyed or not. The forms were collected at the end of two hours and the results were analyzed.

## 3. Results

**Figure 1:**
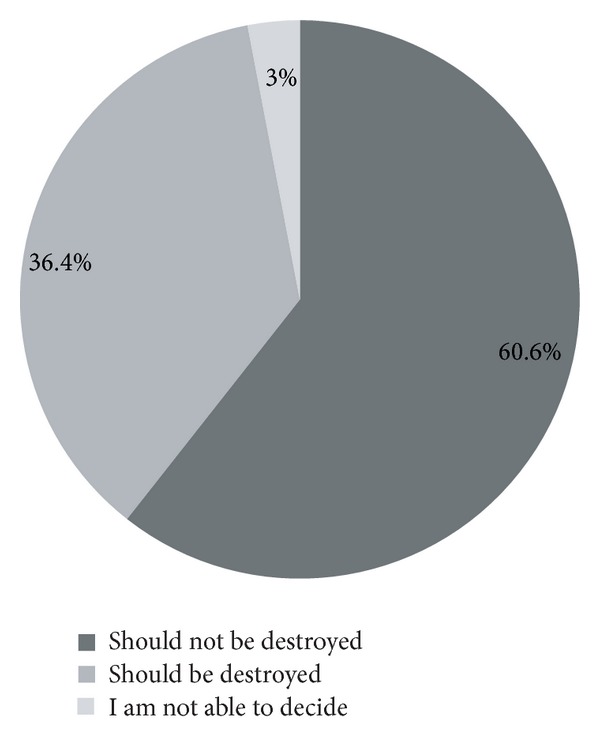
The verdict: to destroy the virus or not. The Pie diagram depicts the opinions of participants on the destruction of remaining variola virus stocks. Majority voted in favour of continued preservation (60.6%).

It is to be noted that 90% of the participants who answered the questionnaire were born in the posteradication era of smallpox. Among the 200 participants, only sixty-six participants had answered the survey. Out of 66, forty (60.6%) had answered that the virus stocks should not be destroyed and twenty-four of them (36.4%) answered that, the virus stocks should be destroyed. The remaining two (3%) had answered that they were not able to decide (Figure 1). It should be noted that 75.75% of the 66 participants who had answered the questionnaire were students. The 66 participants who had answered the questionnaire fairly represented the meeting participants because 28.78% of these 66 participants were from the field of medicine, 63.63% were from biotechnology, 4.54% were from dentistry, and 3.03% were from veterinary sciences.

Among the 60.6% of those participants who answered the questionnaire in favour of the virus stocks not to be destroyed, the major reason quoted (Table 2) was that the virus stocks can be used for future research. Among the 36.4% of the participants who answered the questionnaire that the virus stocks should be destroyed, the major reason quoted was the threat of bioterrorism if the virus stocks are preserved.

## 4. Discussion

This inquiry provides an analysis of important issues from public health, clinical, and research aspects. The verdict is quite strong with nearly 60.6% of those who have answered the question, in favour of preserving the two remaining smallpox viral stocks, and, among the various reasons given, utility of specimens for future research is predominantly strong. Some other minor reasons include historical links. 

Among those who have said it should be destroyed, the major concern was bioterrorism, and they argued that the availability of the full genome of the variola virus and availability of related strains of viruses for study can compensate the loss of the smallpox virus stocks. The other factors include the nonavailability of adequate vaccine stocks and the lack of expertise in prompt diagnosis, treatment, and further control of spread. The current vaccine stock available with the WHO is only about 0.5 million doses [11], which is insufficient by any stretch of imagination to control an outbreak. It is also a fact that most of the third world nations do not have sufficient access to vaccines in case of an unexpected outbreak. What is worse is that there are no back-up laboratories to produce vaccines immediately on a mass scale if any need arises. Moreover, we need trained personnel to administer the latest vaccine to the population, which will be difficult in case of a mass outbreak. Besides, there have been reports of several adverse effects in laboratory studies of the virus [12]. The last reported fatal case of smallpox was that of the medical photographer Janet Parker at the Birmingham Medical School [13]. Judging from this laboratory outbreak of smallpox, the risk of accidental outbreaks in the future from stock piles is evident. Technological advancements which had led to the increase in speed and frequency of air travel by people throughout the world may result in faster spread of smallpox or any other virus in the event of an outbreak such as SARS-CoV [14]. However a heartening fact is that, since smallpox is relatively a slow spreading disease compared to SARS, air travel though could increase the number and distribution of sites with active cases, but the actual human-human spread kinetics may still be slow. Huggins in 1995 found that a drug called cidofovir can block smallpox replication, and he along with Jahrling had believed in around 1999 that in 5-year time, better antismallpox drugs were likely to be discovered. They argued that they had to test the new drugs on the live variola major virus to obtain Food and Drug Administration (FDA) approval and hence virus stocks have to be preserved [15]. The counter arguments to this point of view are that there currently exists at least two compounds which are considered as remedial measures for smallpox such as ST-246 [16] and CMX001 [17]. Moreover, the fully sequenced genome of smallpox virus is also available [18], which can be used to recreate the virus for laboratory drug testing. “Also any likely use of drugs or vaccines against smallpox in case of an outbreak would fall under emergency regulatory provisions that allow the use of treatments that have not been completely tested,” argue a few scientists [19].

The factors in favour of continued preservation of stocks include requirement of an authentic source of naturally occurring virus for research and the fact that smallpox virus is not the only tool for bioterrorism.

Research in biology for developing newer vaccines, newer antivirus drugs, and diagnostic tests against smallpox requires appropriate live strain of viruses, because the viruses artificially reconstructed in the laboratory may or may not exactly recapitulate the biological characteristic of the virus in its native form. For instance, the mutations occurring in a specific strain of virus accumulated over prolonged periods of time might not be possible to be recapitulated in the laboratory in a relatively shorter period of time as a synthetic variola virus (i.e., a clone) would or would not exhibit the same virulence as the “wild” uncloned stocks. Even if recapitulated, the exact biologic extrapolation may not be accurate. Also there are not many studies on the validation of the results of antiviral studies done in viruses in their native form versus their sequenced genome forms in biological models. It has not yet been possible to successfully create animal models of smallpox because variola does not affect non-human primates. Though animal models of other strains of pox viruses, for example, monkey pox, are available and even late stages of smallpox can be modeled in macaques [20], they are still imperfect models compared to an animal model infected with the native strain of the virus exhibiting the disease in its full form. Also, the reason behind why smallpox is so lethal to humans whereas it does not affect animals and the immunological mechanisms behind immunity conferred by the current vaccines all need to be answered by research for which maintenance of the live form of the virus is essential. Intentions to cause bioterrorism not only depend on the presently stored stocks of smallpox virus, but it is possible to recreate deadly virus strains even from related species of smallpox or even from the scratch using today's technology. Even worse is the fact that there is an increased prevalence of infections due to orthopoxviruses like monkey pox and cow pox ever since smallpox was eradicated and vaccination was stopped [21, 22]. Recent controversies on laboratory-bred Avian Influenza viruses, in which mutant strains were developed by scientists [23], throw light on the fact that there are more deadly viruses which can cause accidental outbreaks and can be used for bioterrorism than variola virus. In addition, whether the lab research on such highly dangerous mutant strains should be published or not remains a controversy. Once published, those protocols also have a potential to be misused to develop bioterrorism weapons [24, 25]. This further strengthens the argument that destruction of smallpox virus stocks is not the ultimate solution to the threat of bioterrorism which is the major argument of proponents for destruction of the virus stocks.

It should be pointed out that the meeting is a unique one which has brought together the clinicians and basic scientists, in life sciences together, under one roof, and it is known that a cultural divide between clinicians and basic scientists exists all over the world [26]. This unique gathering is of significant importance to this subject because while the clinicians would have seen or understood the implications of the spread of such viruses from the patient-management point of view, the basic scientists on the other hand would have seen it from the laboratory perspectives. The basic scientists would have understood the difficulties to recreate the virus artificially after destroying the natural virus albeit the recent synthetic biology technologies can use chemically synthesized DNAs to produce a pathogen in the laboratory [27]. This could be one of the explanations to the results wherein most of the basic scientists have opted for preserving the organism while among the clinicians it is a mixed reaction.

**Figure 2:**
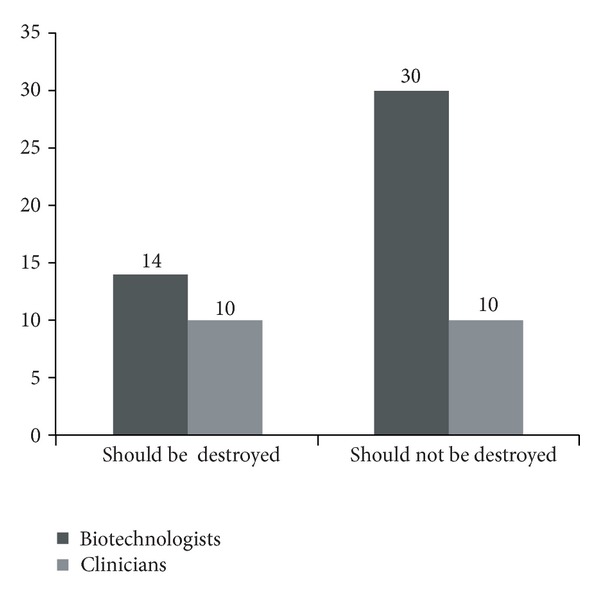
Participants' professional background. The bar chart depicts the professional background of participants who answered the various categories on variola virus stocks destruction. Clinicians were equally divided in their opinion on destruction (50%) and preservation (50%) of the remaining variola virus stocks. Majority of the basic science researchers voted for preservation of the variola virus stocks (68.18%).

The above mentioned assumptions are compatible with the results in this study, as the clinicians are divided equally in their opinion as 50% of them having supported that the variola stocks should be destroyed and the remaining 50% having supported their continued preservation (Figure 2), whereas two-thirds of the basic scientists have supported the continuous preservation of the stocks for future research (Figure 2). There are mainly two concerns—one is the fear of bioterrorism and the other is the fear of losing a valuable natural resource for research. The authors are of a view that strict rules and systems for enforcement should be in place against the creation or handling of potentially life threatening organisms to ensure safety of all living beings in the universal environment, while, in permitted and appropriately guarded laboratories, the naturally created organisms are kept alive for future research. The proportion of the students among the participants was 74.31% and the same reflected in the proportion of the respondents as well (75.75%), that is, 50 out of 66 respondents. In terms of relevance of the results, if majority of the respondents were senior scientists and physicians instead of being students as in this case, it might be considered carrying a relatively higher significance. However, we would like to state that the implications of this question were thoroughly explained by an expert for a better understanding by the respondents before they answered the questions. The results of the study thus may be considered as the opinion of budding scientists representing the younger generation.

## 5. Conclusion

Analyzing the previous facts both in favor as well as against preservation of smallpox virus stocks, the authors stand in line with the verdict of the majority that the variola (smallpox) virus need to be preserved for future biological research as well as vaccine preparation to combat future epidemic.

## Figures and Tables

**Table 1:** Questionnaire given to the participants of the inquiry on the option of preserving/destroying the variola virus.

Submit your thoughts/position/suasions/opinions/beliefs/ideas on this Dynamite Question in the form given below	
Age	
Sex	
Email Id	
Qualification	
Country/location	
Profession	
My Stand on this issue	
Yes the last two stocks of smallpox viruses should be destroyed	
No the last two stocks of smallpox viruses should not be destroyed	
Explanation for my stand in this issue (not exceeding 500 words)	

**Table 2:** Major reasons given by participants to preserve/not to preserve variola virus.

Main reasons favoring preservation (*n* = 40; 60.6%)	(1) For further studies, if mutants evolve, we need the original strains for comparison
	(2) For development of vaccines or antiviral drugs in case of accidental outbreak of the disease
	(3) Historical reasons for preservation of the viruses to serve as a study material for the future generations
	(4) There are many dangerous viruses which can also be misused. Therefore there is no harm in preserving the last two stocks of smallpox

Main reasons against preservation (*n* = 24; 36.4%)	(1) Fear of misuse and bioterrorism
	(2) Genetically modified or lab-bred strains will be difficult to curtail if there is an outbreak due to accidental or deliberate exposure to the stock virus
	(3) Storage is unnecessary without any immediate application of the viruses
	(4) Since the full genome of the smallpox is available, live stocks are not needed
	(5) Other similar viruses which are available can be used for development of drugs against smallpox; hence live smallpox viruses need not be preserved
